# Identification and characterization of microsatellite markers for population genetic studies of *Panstrongylus megistus* (Burmeister, 1835) (Triatominae: Reduviidae)

**DOI:** 10.1186/s13071-021-04771-w

**Published:** 2021-05-22

**Authors:** Flávio Campos Ferreira, Leilane Oliveira Gonçalves, Jeronimo Conceição Ruiz, Leonardo Barbosa Koerich, Fabiano Sviatopolk Mirsky Pais, Lileia Gonçalves Diotaiuti, Carlota Josefovicz Belisário

**Affiliations:** 1 Laboratory of Triatomíneos, Institute René Rachou/FIOCRUZ-MG, Belo Horizonte, Brazil; 2 Biosystems Bioinformatics Group, Institute René Rachou/FIOCRUZ-MG, Belo Horizonte, Brazil; 3 Laboratory of Hematophagous Insect Physiology, Institute of Biological Sciences, Federal University of Minas Gerais, Belo Horizonte, Brazil; 4 Bioinformatics Platform RPT04B, Institute René Rachou/FIOCRUZ-MG, Belo Horizonte, Brazil

**Keywords:** *Panstrongylus megistus*, Microsatellites, *Triatominae*, Chagas Disease

## Abstract

**Background:**

*Panstrongylus megistus* is the most important vector of Chagas disease in Brazil. Studies show that the principal factor hindering the control of triatomines is reinfestation of houses previously treated with insecticides. Studies at the microgeographic level are therefore necessary to better understand these events. However, an efficient molecular marker is not yet available for carrying out such analyses in this species. The aim of the present study was to identify and characterize microsatellite loci for future population genetic studies of *P. megistus*.

**Methods:**

This study work consisted of five stages: (i) sequencing of genomic DNA; (ii) assembly and selection of contigs containing microsatellites; (iii) validation of amplification and evaluation of polymorphic loci; (iv) standardization of the polymorphic loci; and (v) verification of cross-amplification with other triatomine species.

**Results:**

Sequencing of males and females generated 7,908,463 contigs with a total length of 2,043,422,613 bp. A total of 2,043,690 regions with microsatellites in 1,441,091 contigs were obtained, with mononucleotide repeats being the most abundant class. From a panel of 96 loci it was possible to visualize polymorphisms in 64.55% of the loci. Of the 20 loci genotyped, the number of alleles varied from two to nine with an average of 4.9. Cross-amplification with other species of triatomines was observed in 13 of the loci.

**Conclusions:**

Due to the high number of alleles encountered, polymorphism and the capacity to amplify from geographically distant populations, the microsatellites described here show promise for utilization in population genetic studies of *P. megistus*.

**Graphic abstract:**

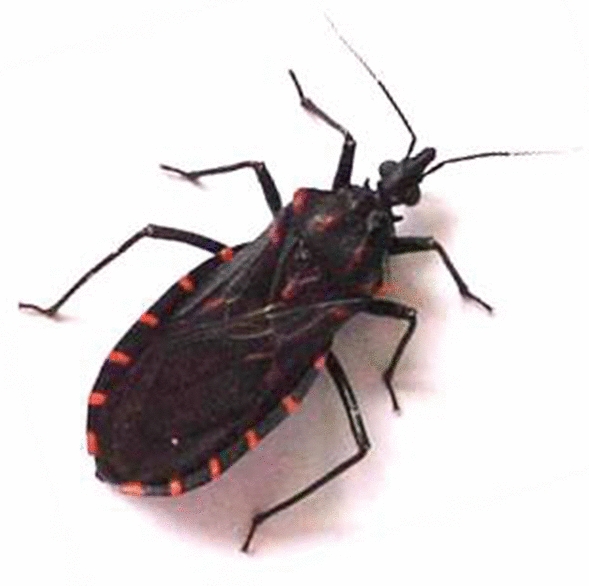

**Supplementary Information:**

The online version contains supplementary material available at 10.1186/s13071-021-04771-w.

## Background

Triatomines are hematophagous insect vectors of *Trypanosoma cruzi* (Chagas, 1909), the etiological agent of Chagas disease, which is considered the principal parasitic disease of Latin America in terms of economic impact and public health [1–3].

Among the autochthonous triatomines of Brazil, *Panstrongylus megistus* (Burmeister, 1835) is considered the vector of greatest epidemiological importance of Chagas disease due to its high capacity to invade and colonize domestic buildings, high levels of infection with *T. cruzi*, eclectic blood-feeding and a wide geographical distribution in Brazil, ranging from the state of Maranhão to the state of Rio Grande do Sul [4–7]. In recent years, the presence of *P. megistus* has also been reported in the Brazialn states of Acre and Rondônia [8].

It has become apparent that the principal factor hindering the control of triatomines is reinfestation of houses previously treated with insecticides, possibly due to insects that survive spraying (residual foci) or triatomines from sylvatic foci [9]. Analysis of this problem requires an investigation at the microgeographic level. Microsatellite molecular markers have been used with success to evaluate the gene flow of triatomine populations between natural and artificial environments (intradomestic and peridomestic) [10–22]. It has been reported that populations of *T. infestans* originating from three Bolivian Andean locations did not show any difference in preference for insects in the artificial environment and those in the natural environment at the same location, suggesting that the source of infestation in the dwellings is wild insects [15]. De Rosas et al. [12] demonstrated that the level of genetic structure of Argentine *T. infestans* populations is approximately 400 m, indicating the possibility of passive dispersion within that distance. To reduce the odds of reinfestation, these authors suggested that insecticide treatment should occur on a larger radius around the infested area. In northern Argentina, an evaluation of the genetic structure of *T. infestans* populations revealed putative sources of reinfestation and its dynamics [22]. These studies illustrate that microsatellites can be useful for understanding the factors that favor the infestation/reinfestation of domiciles.

Given the absence of tools capable of capturing at a fine-scale the process of (re)colonization of *P. megistus* in the artificial environment, the aim of this work was to isolate and characterize microsatellite loci from *P. megistus*, using Illumina HiSeq genome sequencing.

## Methods

This work was perfomed in five stages: (i) genome sequencing of *P. megistus*; (ii) assembly and selection of contigs with microsatellites; (iii) validation of the amplification and evaluation of the polymorphisms in the loci; (iv) standardization of the polymorphic loci; and (v) verification of cross-amplification with other triatomines species.

### Sequencing, identification of regions with microsatellites and development of the primers

Two pools of five *P. megistus* were used, one of female individuals and another of males, from a colony derived from the municipality of Santana do Riacho, Minas Gerais, Brazil (19°10′8″S, 43°42′50″W) maintained in the insectary of the Laboratório de Referência em Triatomíneos e Epidemiologia da Doença de Chagas were selected for sequencing. DNA was extracted from the reproductive organs of the insects using the Genomic DNA Extraction and Purification Kit® (Promega, Madison, WI, USA) following the protocol described by the manufacturer. The libraries were constructed using the TruSeq DNA PCR Free 350 bp kit (Macrogen Inc., Seoul, South Korea) according to the manufacturer’s instructions, and the sequencing of each pool was performed separately using the Illumina HiSeq X platform (Illumina, Inc., San Diego, CA, USA). The raw sequence reads were evaluated in terms of read quality with PRINSEQ [23]. Data filtering and trimming (adaptor removal and Phred quality score cut-off ≥ 25) were performed with Trimmomatic [24]. Contigs were assembled* de novo* with a kmer size of 77 using SOAPdenovo2 [25]. Microsatellite regions were identified with MISA software [26] with the following parameters: minimum of ten repeats of 1 base, six repeats of 2 bases, five repeats of 3 bases, five repeats of 4 bases, five repeats of 5 bases and five repeats with 6 bases each. Microsatellites were selected based on: (i) minimum of six repeats of perfect, di- or trinucleotides; and (ii) identified in contigs with a minimum size of 2 kb. Additionally, Primer3 [27, 28] was used in order to identify primer annealing sites flanking the repeat regions. Only intergenic regions with a predicted amplicon size of between 150 and 400 bp were selected for further analysis.

### Standardization of amplification and evaluation of polymorphism in microsatellite regions

Amplification and polymorphism of the loci selected were evaluated in six specimens of *P. megistus*: (i) two from a colony derived from the municipality of Juquiá, São Paulo, Brazil (24°19′15″S, 47°38′6″W); (ii) two from a colony formed from insects captured in diverse localities in the state of Minas Gerais, Brazil; and (iii) two others from the same colony used for genome sequencing, which came from Santana do Riacho.

The DNA was extracted from two legs of each insect following an adapted protocol of the Wizard® Genomic DNA Purification Kit (Promega) [29]. The DNA was quantified using a NanoDrop One Microvolume UV–Vis spectrophotometer (Thermo Fisher Scientific, Waltham, MA, USA) and stored at − 20 ºC until used.

In order to standardize and optimize the specificity of the PCR amplification, different dilutions of enzymes and cofactors, as well different annealing temperatures (range: 54 °C to 65 °C), were tested. The PCRs were performed in a final volume of 10 µl containing the Master Mix GoTaq Green (Promega), 10 nmol of forward primer, 10 nmol of the reverse primer and approximately 10 ng of template DNA. The reactions were performed on a Veriti thermocycler (Applied Biosystems, Foster City, CA, USA) under the following conditions: an initial denaturation at 95 °C, 4 min; followed by 95 °C/30 s, 54 °C to 65 °C/30 s, 72 °C/30 s for 35 cycles; and a final extension at 72 °C/5 min. The amplicons were run on 8% polyacrylamide gels stained with silver nitrate. The approximate size of the fragments was determined using the molecular marker ΦX 174 DNA HaeIII (Promega).

### Characterization of polymorphic microsatellite loci

Among the evaluated loci that were found to be polymorphic under the conditions described in the previous section, 20 were selected for standardization (Table 1) based on an amplicon size of between 100 and 300 bp and annealing temperature, for use in future multiplex PCRs.

**Table 1:** Primer sequence and repeat motif of the genotyped microsatellite loci

Locus	Primer Sequence 5'–3'	Repeat motif
Pm002	F: CACACAGAGGCGATTCGGTA	(TA)_8_
	R: GTCTGCTGCCGCAATTTCTC	
Pm008	F: AAAACCACAGGAAGCTCGAA	(CA)_6_
	R: GTCTTCAGCTCCGGTCATGC	
Pm015	F: TGTACCCTATATAACGCGCCA	(AG)_7_
	R: ACATCTAAGCCCTTAGTGCGA	
Pm018	F: TGAACAAAGCTACCTGGAAAAGC	(AT)_7_
	R: ACAAGGATCCTGGGAAAGCG	
Pm027	F: TGTGGATACTTAGGGCATAGCA	(TA)_15_
	R: ACGATGTGTGAAAATTAGAGCAACA	
Pm030	F: ATCCCATGCGTCCCAATAGC	(AT)_7_
	R: TCCGAGAAAAAGTCGTTATCCA	
Pm044	F: ATCTTCGGAATCCCTGACGC	(TG)_6_
	R: AGTTTGAGAACTTCCTGCGGT	
Pm048	F: GCTGGCCAGAAGTCCCTTTA	(AC)_8_
	R: ACCAAGTCTGACCACTTCTTTCT	
Pm049	F: TCCGATCACCAAATGTGCGA	(TG)_6_
	R: CAGCCACTTAGTGAACCCCC	
Pm051	F: CCTTTGGATAGCGCAGGGTT	(AAT)_5_
	R: TCAAAGGCACCCGTTGAAGT	
Pm054	F: TCGGCAACAGTACTCAACGA	(AAT)_8_
	R: TCCTTTATGAGTAAACGGCGTGA	
Pm055	F: TGAATGTGGAGCGAATGTGA	(ATT)_5_
	R: AGCATCTCCTCTGACGGTCT	
Pm058	F: AGTATCGTCCCTGCAGCCTA	(TAT)_6_
	R: ACAACGGCAGAATTAACTTCCA	
Pm063	F: TGGGTTTTCGTAGTATCTTTCCCA	(TTA)_8_
	R: ACCAGAATTATGACAGTAGAGCGT	
Pm066	F: ACACGACTTTCTCTTACTCCTGT	(GTG)_5_
	R: GTGAGCTCTACTGCGTCACA	
Pm071	F: TGTGGACTGGTCTTGGGAAA	(TAC)_5_
	R: GGGGGTGGGAATAAAAGCCT	
Pm076	F: TGCGAGATTGAATTTGCGAGA	(ATA)_6_
	R: TGCTCTCTTAGGGCCTGTCT	
Pm079	F: TGTCCGAGCTCTCCCAGAAT	(GAA)_5_
	R: TACCTCAGCCCAGGAAGGTT	
Pm081	F: CCCACACACACACACCCATA	(ATT)_6_
	R: ACTCCGCTTTCTAGTGTGAGC	
Pm083	F: TTTCGCCTCTGCCCAAGAAT	(AAT)_8_
	R: AGAGAAATGGGCACACCTGG	

Fifteen specimens of *P. megistus* captured in the municipality of Jaboticatubas, Minas Gerais, Brazil (19°30′50″S, 43°44′42″W) by Belisário et al. [30] were used. These samples were divided into two groups in order to evaluate intra-populational variability: nine insects from the locality Fazenda Santo Antônio (group I); and one insect each from the following localities: Barreiro do Papagaio, Fazenda Espada, Capão Grande II, Fazenda Borges, Guarazinho, and Fazenda Boiça (group II). The second group also included the insects from Santana do Riacho and Juquiá described in section Standardization of amplification and evaluation of polymorphism in microsatellite regions.

Four other species of triatomines were used to evaluate cross-amplification: (i) one specimen of *Panstrongylus diasi* Pinto & Lent 1946 from the insect collection of the Instituto René Rachou Fiocruz Minas/Belo Horizonte, Brazil; (ii) one specimen of *Panstrongylus lignarius* (Walker, 1873); (iii) one specimen of *Triatoma tibiamaculata* (Pinto, 1926); and (iv) one specimen of *Triatoma sordida* (Stal 1859). These last three insects were derived from the colonies of the Laboratório de Referência em Triatomíneos e Epidemiologia da Doença de Chagas of the Instituto René Rachou, Fiocruz Minas.

The DNA was extracted from two legs from each individual as described in section Standardization of amplification and evaluation of polymorphism in microsatellite regions. The PCRs were performed in a total final volume of 10 µl containing 5× Colorless GoTaq® Flexi (Promega), 3 mM MgCl_2_, 10 nmol of the fluorescently-labeled forward primer, 10 nmol of the reverse primer and approximately 10 ng of template DNA. The reactions were performed in a Veriti® 96-well thermocycler (Applied Biosystems) using the following cycling conditions: an initial denaturation at 95 °C, 4 min; then 95 °C/30 s, 65 °C/ 30 s, 72 °C/30 s for 35 cycles; 95 °C/30 s, 53 °C/ 30 s, 65 °C/30 s, 72 °C/30 s for 3 cycles; and a final extension of 72 °C for 5 min. The products were diluted in the ratio of 1:10 in UltraPure Distilled Water (Invitrogen, Carlsbad, CA, USA) and genotyped on an ABI 3730 Sequencer (Life Technologies, Carlsbad, CA, USA) using the size standard GeneScan Liz 500 of the sequencing platform of Sequenciamento de DNA por Eletroforese Capilar of the Instituto René Rachou. The chromatograms were analyzed using the software Geneious (ver. 10.1.3) [31]. The number of alleles with observed heterozygosity (OH) and expected heterozygosity (EH) (Arlequin ver. 3.5.2.2) [32] and the presence of null alleles (MICRO-CHEKER ver. 2.2.3) [33] were evaluated.

## Results

Sequencing of the *P. megistus* genome yielded 463,151,518 reads (90.41% > Q30) for the female pool and 338,531,204 reads (89.54% > Q30) for the male pool. The GC-content was 34.53 and 35.06%, respectively. The* de novo* assembly was made using only reads with a quality > 25, generating a total of 7,908,463 contigs with a total length of 2,043,422,613 bp. The N50 (sequence length of the shortest contig at 50% of the total genome length) of the assembly was 1034 and the final GC-content was 33.14%. We identified a total of 2,043,690 microsatellite regions located in 1,441,091 contigs with mononucleotide repeats being the most abundant (1,054,968, corresponding to 51.62%) and pentanucleotide repeats present at a lower quantity (1337, corresponding to 0.06%). In accordance with the parameters described in the Methods section, 79 different microsatellite regions were selected for further analysis.

Among the 96 primer pairs selected for PCR amplification, 79 resulted in amplicons visualized on polyacrylamide gels after standardization. The annealing temperature after standardization varied from 60 °C to 67 °C (Additional file 1: Table S1). It was possible to visualize the presence of polymorphism on the gel for 64.55% (51) of the loci. Among these 51 loci, 20 markers were selected for characterization (Table 1). It was not possible to standardize the amplification of 17 primer pairs due either to low specificity or the absence of amplicons.

In the samples of *P. megistus*, the number of alleles per locus varied from two (*Pm051*, *Pm071* and *Pm079*) to nine (*Pm049*), with a mean of 4.9 (Table 2). Among the 20 loci genotyped, two (*Pm051* in group II and *Pm058* in group I) were monomorphic and for one (*Pm054*) it was not possible to visualize peaks on the chromatogram (Table 3).

**Table 2:** Allele number and size range per loci for each triatominae species used in the study

*Locus*	*Panstrongylus megistus*		*Panstrongylus lignarius*		*Panstrongylus diasi*		*Triatoma tibiamaculata*		*Triatoma sordida*	
	AN	SR	AN	SR	AN	SR	AN	SR	AN	SR
Pm002	8	122–138	0	–	0	–	0	–	0	–
Pm008	3	268–276	1	168	0	–	0	–	0	–
Pm015	7	274–286	0	–	0	–	0	–	0	–
Pm018	4	204–210	1	192	0	–	1	194	1	194
Pm027	8	170–192	0	–	1	188	2	166–178	1	114
Pm030	6	196–218	1	178	1	88	1	174	1	84
Pm044	6	218–248	1	86	1	86	0	–	0	–
Pm048	7	246–268	0	–	0	–	0	–	0	–
Pm049	9	268–294	0	–	0	–	2	155–181	1	80
Pm051	2	247–250	0	–	0	–	0	–	0	–
Pm055	3	193–199	0	–	0	–	0	–	0	–
Pm058	3	270–276	1	82	0	–	1	114	1	170
Pm063	5	269–284	0	–	0	–	2	234–246	0	–
Pm066	3	162–174	2	151– 162	0	–	0	–	1	176
Pm071	2	162–165	0	–	0	–	0	–	0	–
Pm076	3	234–240	0	–	1	234	0	–	0	–
Pm079	2	275–278	1	180	0	–	1	74	1	74
Pm081	5	169–181	0	–	0	–	1	210	0	–
Pm083	8	237–267	0	–	0	–	1	75	1	81
Mean	4.847		0.421		0.210		0.631		0.421	

*AN* Allele number,* SR* size range

**Table 3:** Number of genetic copies, allele number, observed heterozygosity and expected for each *Panstrongylus megistus* group

Locus	Group I^a^				Group II^b^			
	GC	AN	OH	EH	GC	AN	OH	EH
Pm002	18	4	0.11111	0.68627	20	7	0.10000	0.85789
Pm008	18	2	0.00000	0.52288	20	3	0.00000	0.67368
Pm015	18	4	0.44444*	0.72549*	20	7	0.50000	0.82105
Pm018	18	4	0.33333	0.75817	20	4	0.40000*	0.60000*
Pm027	18	4	0.55556*	0.69935*	14	7	0.28571	0.87912
Pm030	18	3	0.22222	0.52288	20	6	0.40000	0.75789
Pm044	18	4	0.33333	0.66013	20	5	0.40000	0.72632
Pm048	18	6	0.33333	0.73856	20	5	0.20000	0.78947
Pm049	18	6	0.55556*	0.74510*	20	7	0.60000*	0.88421*
Pm051	18	2	0.00000	0.47059	20	This *locus* is monomorphic		
Pm055	18	3	0.44444*	0.58170*	20	2	0.00000	0.50526
Pm058	16	This locus is monomorphic			20	3	0.20000*	0.35263
Pm063	18	2	0.33333*	0.52941*	20	5	0.50000	0.74211
Pm066	18	3	0.44444*	0.38562*	20	2	0.00000*	0.18947*
Pm071	18	2	0.22222	0.20915*	20	2	0.10000*	0.10000*
Pm076	18	2	0.00000	0.47059	16	3	0.12500	0.49167
Pm079	18	2	0.00000	0.36601	20	2	0.00000	0.44211
Pm081	18	5	0.44444*	0.55556*	20	3	0.40000	0.46842
Pm083	18	5	0.55556*	0.77778*	20	6	0.20000	0.77895
Mean	18.000	3.500	0.29630	0.57807	19.444	4.389	0.24504	0.61446
s.d	0.000	1.383	0.20166	0.15924	1.653	1.944	0.19651	0.23607

*GC *Genetic copies,* OH* observed heterozygosity,* EH* expected heterozygosity

*Significant value at  *P* < 0.05

^a^Group I: nine insects from Fazenda Santo Antônio; Group II: one insect from each of the following locations: Barreiro do Papagaio, Fazenda Espada, Capão Grande II, Fazenda Borges, Guarazinho and Fazenda Boiça (all localities in the municipality of Jaboticatubas, MG); and two insects from the municipality of Santana do Riacho, MG and municipality of Juquiá, SP

The OH of group I varied from 0.00000 (*Pm002*) to 0.55556 (*Pm049*) (mean 0.29630), while the EH varied from 0.20915 (*Pm071*) to 0.77778 (*Pm083*) (mean 0.57807). In group II, the OH varied from 0.00000 (*Pm002*) to 0.60000 (*Pm049*) (mean 0.24504), while the EH varied from 0.10000 (*Pm071*) to 0.88421 (*Pm049*) (mean 0.61446) (Table 3).

Of the 20 loci analyzed, group I had null alleles at seven loci (*Pm002*, *Pm008*, *Pm018*, *Pm048*, *Pm051*, *Pm076* and *Pm079*) and group II had null alleles at five loci (*Pm002*, *Pm027*, *Pm048*, *Pm079* and *Pm083*).

Regarding cross-amplification, 13 loci were amplified from other species. The *Pm030* marker was amplified from all the samples tested. However, the number of alleles was lower than that in *P. megistus*, varying from one to four, with a mean of 1.5 (Table 2). OH and EH were not calculated because there was only one specimen for each of the species evaluated.

## Discussion

This study is novel in two aspects: in the development of primers to microsatellites of *P. megistus* and in the methodology used. Unlike previously published studies on the identification of microsatellite markers in triatomines, in which the methodology most commonly used is enriched libraries and the subsequent use of probes for the selection of microsatellite regions [34–40], the present study is the first to use next-generation sequencing. This methodology was chosen due to the limited information available on the genome of *P. megistus*, the principal species transmitting *T. cruzi* in Brazil.

For the design of the initial primers, we chose to synthesize only primers to regions flanking dinucleotide and trinucleotide repeats because these latter arrangements have higher mutation rates compared to other microsatellite classes [41]. Therefore, they will be more informative in future population genetic studies.

To make the PCR reactions more specific, we tested different annealing temperatures. The optimal temperatures utilized were those observed prior to the absence of bands in the polyacrylamide gel, as well as prior to the dilution of enzymes and cofactors. Modification of such determinants can alter the success of PCRs [42]. Even after these attempts, it was not possible to standardize the PCR for 17.7% of the 96 loci selected due to the absence of fragments or the presence of nonspecific fragments. In these cases, the primer may have been designed to a non-conserved region or have more than one binding site.

Two loci (*Pm051* in group II and *Pm058* in group I) exhibited monomorphic patterns and in another locus (*Pm054*) it was not possible to visualize peaks in the chromatogram. However, working with a larger sample number can increase the chances of observing heterozygous and polymorphic individuals [43]. Accordingly, these three loci will be evaluated in a larger sample set in a future study to verify the absence of polymorphism, since it was possible to visualize amplicons from these loci in polyacrylamide gels.

The quantity of alleles encountered in the 20 loci tested differed from that that encountered in other studies on triatomines: in *T. dimidiata*, the mean allele number (AN) was reported to be 16, varying from six to 27 alleles at eight loci [35]; in *T. infestans*, the mean AN was 9.7, varying from five to 17 alleles at 13 *loci* [36]; in *Rhodnius pallescens*, the mean observed AN was nine alleles, varying from two to 20 at ten loci analyzed [34]; in *T. sordida*, the mean AN was 7.4, varying from one to 12 at ten loci [44]; in *T. pseudomaculata*, the mean AN was 6.6, varying from two to 15 alleles at seven loci [39]; and for *T. brasiliensis*, the mean observed AN was five at seven standardized loci, ranging from one to 13 alleles per locus [40]. This difference can be explained by the sample size in each study, ranging from 34 [35] to 171 [44] samples, while we used 19 samples of *P. megistus*.

The presence of four loci in* P. diasi* and eight in *P. lignarius* was expected due to the conservation of some genomic regions in phylogenetically related species. This has also been reported in other studies [35, 36, 38–40, 44]. However, the present work is the first to report amplification in species from different genera (*T. sordida* and *T. tibiamaculata*).

The values observed for OH and EH may be indicative of the presence of excessive homozygotes in the groups tested, population structure or the presence of null alleles [45]. The analysis of null alleles demonstrated low occurrence in both groups (35 and 25% in groups I and I, respectively). The presence of null alleles can occur due to mutations in the flanking sequences of the microsatellite regions, thus preventing binding of the primers. The regions not amplified result in apparently homozygous samples when present in a heterozygous state [46, 47].

In the last 40 years, microsatellites have been the most used molecular marker to access polymorphisms of a wide variety of organisms. One of the difficulties in applying this methodology is the need for prior knowledge of the genome. Currently, this problem can be overcome by using the genotyping-by-sequencing (GBS) as a marker. However, GBS requires a greater amount of DNA, throughput and the cost is higher [48].

## Conclusions

Due to the high polymorphism and number of alleles encountered in each locus, as well as the capacity to amplify from geographically distant populations, we conclude that the markers developed in this study show promise for population genetic studies of *P. megistus.* Thus, we hope to help to elucidate the reinfestation processes in the artificial environment by this vector.

## Supplementary Information

**Additional file 1: Table S1.** Forward (F) and reverse (R) primer sequences, repeat motif, annealing temperature (AT) and presence (+) or absence (−) of polymorphism of the* Panstrongylus megistus* microsatellite loci.

## Data Availability

The datasets used and analyzed during the current study are available from the corresponding author.

## Abbreviations

AN: Allele number
EH: Expected heterozygosity
OH: Observed heterozygosity
