# Supplementary material for: Identification and characterization of microsatellite markers for population genetic studies of Panstrongylus megistus (Burmeister, 1835) (Triatominae: Reduviidae)
Source: Parasit Vectors. 2021 May 22;14:273. doi: 10.1186/s13071-021-04771-w (PMC8140489; doi:10.1186/s13071-021-04771-w)
Supplement: Supplementary file 1 — Additional file 1: Table S1. Forward (F) and reverse (R) primer sequences, repeat motif, annealing temperature (AT) and presence (+) or absence (−) of polymorphism of the Panstrongylus megistus microsatellite loci. [file 13071_2021_4771_MOESM1_ESM.docx]

| Table S1. Forward (F) and reverse (R) primer sequence, repeat motif, annealing temperature (AT) and presence (+) or absence (-) of polymorphism of the Panstrongylus megistus microsatellite loci | | | | | |
| --- | --- | --- | --- | --- | --- |
| *Locus* | Primer sequence 5'-3' | | Motif | AT (ºC) | Polymorphism |
| Pm001 | F: | CAGTGTTCTGGGGATTTTCTCG | (TA)_8_ | 66 | + |
|  | R: | GGAGACCGACCAGCTTGATT |  |  |  |
| Pm002 | F: | CACACAGAGGCGATTCGGTA | (TA)_8_ | 65 | + |
|  | R: | GTCTGCTGCCGCAATTTCTC |  |  |  |
| Pm003 | F: | ATCAACTGGCAAATGGCTGC | (TA)_6_ | 66 | + |
|  | R: | CACGCCCAAACTTTTTGCATT |  |  |  |
| Pm004 | F: | AAGAGCCAAGAGGTTGTGGG | (TA)_7_ | 66 | + |
|  | R: | GAGAGGTGAAACGTCCCTGG |  |  |  |
| Pm005 | F: | CCACCACCCGCTTTTTGTTT | (TA)_8_ | 67 | + |
|  | R: | GCCCACTACCAAACTAGCCA |  |  |  |
| Pm007 | F: | TCGTCAACAATGTAGCCCAGA | (AC)_8_ | 65 | - |
|  | R: | CACTTCTAGAATGGGTGGGCA |  |  |  |
| Pm008 | F: | AAAACCACAGGAAGCTCGAA | (CA)_6_ | 65 | + |
|  | R: | GTCTTCAGCTCCGGTCATGC |  |  |  |
| Pm009 | F: | TTGTGGTCCCTTAGGTCCGA | (GT)_8_ | 64 | + |
|  | R: | CGTTGAAGCCGTTTGTGAGA |  |  |  |
| Pm011 | F: | TTGCTTCCGTAAGAAGAGAGAA | (TA)_8_ | 60 | - |
|  | R: | ACACAGCTCAACTTGGCGAT |  |  |  |
| Pm012 | F: | CCCAGCAGTACATAAGGCGA | (TG)_7_ | 66 | + |
|  | R: | TCCGGTTGAGGTATTTTGTCGT |  |  |  |
| Pm013 | F: | TGGTAGTGAGTGTGTGACCC | (AT)_8_ | 65 | - |
|  | R: | GCTATCAGGGGAAGGTTGGT |  |  |  |
| Pm014 | F: | AGCTTCAGTGGTTCGCCATT | (TA)_9_ | 63 | - |
|  | R: | CATGCATCAAACATTTATATACAGGAC |  |  |  |
| Pm015 | F: | TGTACCCTATATAACGCGCCA | (AG)_7_ | 65 | + |
|  | R: | ACATCTAAGCCCTTAGTGCGA |  |  |  |
| Pm016 | F: | AGTGACTTCTCTTCAGCAAGTG | (TA)_7_ | 64 | + |
|  | R: | TCGTTTTTCAGCCATGCTGTT |  |  |  |
| Pm017 | F: | AAATTATGAGCAAACACCGCTAT | (AT)_8_ | 64 | + |
|  | R: | AGAACAGCCTCAACGAAGCA |  |  |  |
| Pm018 | F: | TGAACAAAGCTACCTGGAAAAGC | (AT)_7_ | 65 | + |
|  | R: | ACAAGGATCCTGGGAAAGCG |  |  |  |
| Pm019 | F: | AGGGGAAGGAGGACATACCC | (GT)_8_ | 65 | - |
|  | R: | TGAGCTTCCTACGATTTTCCGT |  |  |  |
| Pm020 | F: | TGAGGTTCCTGGAGTAGGGAG | (TG)_13_ | 65 | + |
|  | R: | ACCTTCGAGTCAGATGCGAT |  |  |  |
| Pm022 | F: | CCAAGGAGGCATTTTTGGACA | (TA)_6_ | 65 | - |
|  | R: | TGGATAGTTTAAAAGGGGCGGT |  |  |  |
| Pm023 | F: | ACATGTTTTGGGTTCCCCTGA | (GT)_11_ | 65 | + |
|  | R: | AGTTTGAGCTTCCTGCGACA |  |  |  |
| Pm025 | F: | GGCCTGCCTAGCAGAAGAAA | (AT)_6_ | 65 | - |
|  | R: | TCTGCTATCTGGCGACCTCT |  |  |  |
| Pm026 | F: | AGTTGGAGGCAGAAGTTGGG | (TG)_6_ | 66 | + |
|  | R: | GCGTTGTGTTGGTAGCACTG |  |  |  |
| Pm027 | F: | TGTGGATACTTAGGGCATAGCA | (TA)_15_ | 65 | + |
|  | R: | ACGATGTGTGAAAATTAGAGCAACA |  |  |  |
| Pm028 | F: | TTGCATCTCGGTATAAAGCCA | (AG)_7_ | 64 | - |
|  | R: | ACCCAATGTAATTTGGCAGCA |  |  |  |
| Pm029 | F: | ACTGATGAGTGGATTGGTGAGA | (AT)_6_ | 65 | - |
|  | R: | TAAACACCCGATCGGCTTCA |  |  |  |
| Pm030 | F: | ATCCCATGCGTCCCAATAGC | (AT)_7_ | 65 | + |
|  | R: | TCCGAGAAAAAGTCGTTATCCA |  |  |  |
| Pm031 | F: | CGCATCCCCTGATAGAACCC | (AT)_8_ | 67 | - |
|  | R: | GCGATCAATTCTCCGAGAGG |  |  |  |
| Pm032 | F: | GCGAGGTCCTAGACAATGACA | (TC)_8_ | 66 | - |
|  | R: | GGGGGAACCTCCAATTCTGA |  |  |  |
| Pm033 | F: | GTCTAGTAGCGGCTGGCAAT | (AT)_6_ | 65 | - |
|  | R: | GGCCAATTTGGTAGTTCACGA |  |  |  |
| Pm034 | F: | ATCCTCCAAGACGAAAGCCG | (TA)_6_ | 66 | + |
|  | R: | CAAACACACACACCCACACA |  |  |  |
| Pm035 | F: | GAGGTTGCAGTGCATTTGGC | (AC)_8_ | 66 | - |
|  | R: | AGGCAATGATAGGAAACGATCCA |  |  |  |
| Pm036 | F: | TGTATGGCCGTGGTGTTTGT | (AT)_6_ | 64 | - |
|  | R: | TCGTCAATAGCTACAATGATTAGGGA |  |  |  |
| Pm038 | F: | CGCTGTGGAACAACAGTTCG | (TC)_7_ | 65 | + |
|  | R: | TGCACACAAACACACAAATAGA |  |  |  |
| Pm039 | F: | TGAGGCCCTCTGAGTACGAA | (TG)_6_ | 66 | + |
|  | R: | AAGTTGCACTTCCGGTTGGT |  |  |  |
| Pm040 | F: | TCAGTCCAAATGCTTGTCTGT | (TA)_9_ | 65 | - |
|  | R: | CGCCATGTTTGCAGTTTTGTC |  |  |  |
| Pm041 | F: | GAACATCGATCTGTTCGCGT | (TA)_6_ | 67 | + |
|  | R: | GACACAATACTCTGGCGGCT |  |  |  |
| Pm042 | F: | TGGCAAGTTTACAGTTAGCGG | (AG)_7_ | 67 | + |
|  | R: | CGGACCGGAAGTGTGTGTTA |  |  |  |
| Pm043 | F: | TGCAAAAACAACTACCAACATTTCA | (CA)_11_ | 63 | - |
|  | R: | GTGCTAAGCCCTGGACTTGT |  |  |  |
| Pm044 | F: | ATCTTCGGAATCCCTGACGC | (TG)_6_ | 65 | + |
|  | R: | AGTTTGAGAACTTCCTGCGGT |  |  |  |
| Pm045 | F: | TGGGGCTGAGAATTTGGCTT | (GA)_7_ | 65 | - |
|  | R: | TCGAATCCGAAACCTCCGTG |  |  |  |
| Pm047 | F: | AGGTGTTGCTATTGTCACGTC | (CT)_10_ | 65 | + |
|  | R: | TCTCTCGCAGGTTTACGAAATGA |  |  |  |
| Pm048 | F: | GCTGGCCAGAAGTCCCTTTA | (AC)_8_ | 65 | + |
|  | R: | ACCAAGTCTGACCACTTCTTTCT |  |  |  |
| Pm049 | F: | TCCGATCACCAAATGTGCGA | (TG)_6_ | 65 | + |
|  | R: | CAGCCACTTAGTGAACCCCC |  |  |  |
| Pm051 | F: | CCTTTGGATAGCGCAGGGTT | (AAT)_5_ | 65 | + |
|  | R: | TCAAAGGCACCCGTTGAAGT |  |  |  |
| Pm052 | F: | ACGATCGAGTTTTCACCGGA | (TAA)_5_ | 66 | + |
|  | R: | CGGGAGGTCCTAGGGAGTTT |  |  |  |
| Pm053 | F: | TGTTGGCGGGAAAAATTGAAGA | (ATA)_5_ | 66 | - |
|  | R: | GGAGTCCAACGCACTAACCA |  |  |  |
| Pm054 | F: | TCGGCAACAGTACTCAACGA | (AAT)_8_ | 65 | + |
|  | R: | TCCTTTATGAGTAAACGGCGTGA |  |  |  |
| Pm055 | F: | TGAATGTGGAGCGAATGTGA | (ATT)_5_ | 65 | + |
|  | R: | AGCATCTCCTCTGACGGTCT |  |  |  |
| Pm056 | F: | GCTCGCGGAATTATCCCAGT | (ATT)_7_ | 66 | + |
|  | R: | CAACCTGAGCTGGACGTAGG |  |  |  |
| Pm058 | F: | AGTATCGTCCCTGCAGCCTA | (TAT)_6_ | 65 | + |
|  | R: | ACAACGGCAGAATTAACTTCCA |  |  |  |
| Pm060 | F: | GATGAGGTCATGGAGGCGAG | (ATA)_6_ | 67 | + |
|  | R: | CAAAAAGTGGCATCTCGGCA |  |  |  |
| Pm062 | F: | ACCAACTTTGGCAATTCTGAGT | (AAT)_7_ | 66 | + |
|  | R: | GCTGATGCCGTTTGACCAAG |  |  |  |
| Pm063 | F: | TGGGTTTTCGTAGTATCTTTCCCA | (TTA)_8_ | 65 | + |
|  | R: | ACCAGAATTATGACAGTAGAGCGT |  |  |  |
| Pm064 | F: | ATAGGCGCTAATGACCTCGG | (AAT)_8_ | 67 | + |
|  | R: | CCGGTATTGGAGAGACCTGC |  |  |  |
| Pm065 | F: | TGCATGTTGTACGCTGTCAAG | (AAT)_6_ | 65 | - |
|  | R: | TTGCAAGATGGGCTTCCAGT |  |  |  |
| Pm066 | F: | ACACGACTTTCTCTTACTCCTGT | (GTG)_5_ | 65 | + |
|  | R: | GTGAGCTCTACTGCGTCACA |  |  |  |
| Pm067 | F: | ACTGCCTGCACTATCTTCCG | (GAA)_5_ | 65 | - |
|  | R: | TCCTACTGCAAGCGGATAGC |  |  |  |
| Pm069 | F: | AGCACGCACAAGAGACCAAT | (TAA)_6_ | 65 | - |
|  | R: | AAGACTTTCGTTGTGGACTCA |  |  |  |
| Pm070 | F: | AAATCGCGCGGTCTTAGAGT | (AAT)_6_ | 65 | - |
|  | R: | TCACAGTGCATTATCCCCCA |  |  |  |
| Pm071 | F: | TGTGGACTGGTCTTGGGAAA | (TAC)_5_ | 65 | + |
|  | R: | GGGGGTGGGAATAAAAGCCT |  |  |  |
| Pm073 | F: | GCACTGCCATATTGGTGGC | (ATA)_6_ | 64 | - |
|  | R: | AGGAGGAAGCACAGTTTCCA |  |  |  |
| Pm076 | F: | TGCGAGATTGAATTTGCGAGA | (ATA)_6_ | 65 | + |
|  | R: | TGCTCTCTTAGGGCCTGTCT |  |  |  |
| Pm077 | F: | AGGCTCCGAGTGCCAATAAC | (AAT)_5_ | 65 | - |
|  | R: | TTGTACTCCCGTCACCCTCA |  |  |  |
| Pm078 | F: | TCAGAGTAGTCGTTGTTTTCTTGT | (TAT)_5_ | 63 | + |
|  | R: | GGGCCGCATAGACACTTGTT |  |  |  |
| Pm079 | F: | TGTCCGAGCTCTCCCAGAAT | (GAA)_5_ | 65 | + |
|  | R: | TACCTCAGCCCAGGAAGGTT |  |  |  |
| Pm081 | F: | CCCACACACACACACCCATA | (ATT)_6_ | 65 | + |
|  | R: | ACTCCGCTTTCTAGTGTGAGC |  |  |  |
| Pm083 | F: | TTTCGCCTCTGCCCAAGAAT | (AAT)_8_ | 65 | + |
|  | R: | AGAGAAATGGGCACACCTGG |  |  |  |
| Pm084 | F: | ACGAGATGGCTGAATAGCCG | (AAT)_5_ | 64 | - |
|  | R: | GGAGGGCTGACTGATGCAAT |  |  |  |
| Pm085 | F: | TAATTGCCGGATCGGAGAGC | (TGA)_5_ | 67 | + |
|  | R: | GGGCAGGAGTATCCGAGAGA |  |  |  |
| Pm086 | F: | AGCTCTGGCCGTCTATCTTT | (TTA)_6_ | 65 | + |
|  | R: | AGCTATAAGGGGTCCACGGG |  |  |  |
| Pm087 | F: | CAGCTGCTCACTCTCAGTTCA | (TAA)_7_ | 65 | + |
|  | R: | TGCATAAACAACCCCTCGGA |  |  |  |
| Pm088 | F: | CACGTTCTTGGTCCACCCTT | (TAT)_5_ | 64 | + |
|  | R: | TGTCAAAACTGTCGCAATTGTA |  |  |  |
| Pm090 | F: | ACTGCCAGTCCATGATGTAAGA | (TAT)_7_ | 60 | + |
|  | R: | TCTGGAACACTACATGAGCAAT |  |  |  |
| Pm092 | F: | TCCCCAGAGTTTCCCTCGAT | (TTA)_5_ | 65 | - |
|  | R: | GTCCCGTTCAGGAAAACAGC |  |  |  |
| Pm095 | F: | TGTTCCCACACACACATACCA | (ATA)_5_ | 66 | + |
|  | R: | TCCAAAGTGAATGTGGATTTGCC |  |  |  |
| Pm096 | F: | ACCAAACACAAACAAATGATCACA | (TAT)_6_ | 64 | + |
|  | R: | ACCTACGACAAACAATCCCGT |  |  |  |
| Pm098 | F: | TGGACTAGAACCCGGGACTT | (TTA)_5_ | 65 | - |
|  | R: | AGTGTTGCACACCCAATGTG |  |  |  |
| Pm099 | F: | TCCTCTTCCCAGAACCCTCA | (AAT)_5_ | 62 | + |
|  | R: | TCGTTTTTGGCTGGGGGAAT |  |  |  |
| Pm100 | F: | GCGGACGGATTCCTAAGTGT | (TAT)_5_ | 65 | - |
|  | R: | CTCGAACAACCACATTCGTGT |  |  |  |
